# Cognitive tasks elicit mental fatigue and impair subsequent physical task endurance: Effects of task duration and type

**DOI:** 10.1111/psyp.14126

**Published:** 2022-06-21

**Authors:** Neil Dallaway, Samuel J. E. Lucas, Christopher Ring

**Affiliations:** ^1^ School of Sport, Exercise and Rehabilitation Sciences University of Birmingham Birmingham UK

**Keywords:** effort, electromyography, heart rate variability, mental fatigue, muscular fatigue, rate of perceived exertion

## Abstract

Although mentally fatiguing cognitive tasks can impair subsequent physical endurance, the importance of cognitive task duration and the role of response inhibition remain unclear. This study compared the effects of a serial incongruent Stroop color‐classification task (i.e., with response inhibition) and N‐back memory updating task (i.e., without response inhibition) on mental fatigue and subsequent rhythmic handgrip exercise. Participants (*N* = 90) were randomly assigned to one of three cognitive task groups (Stroop, 2‐back, control) and completed four 10‐min blocks of one cognitive task followed by a 5‐min physical endurance task (self‐paced rhythmic handgrip exercise). Heart rate, heart rate variability, electromyographic forearm activity, and force were recorded throughout along with self‐reported measures of fatigue, exertion, and motivation. From the start, the Stroop and 2‐back tasks elicited higher heart rate and lower heart rate variability as well as greater fatigue, effort, and interest/enjoyment than the control task. From the second block onwards, the Stroop and 2‐back groups produced less force than the control group. There were no group differences in forearm muscle activity. In sum, mental fatigue was induced after performing a cognitive task for 10 mins, whereas muscular endurance was impaired after performing a cognitive task for 20 mins. That these effects were observed for both types of cognitive task indicates that response inhibition is not a necessary condition. The cognitive task duration required to induce mental fatigue and impair rhythmic handgrip endurance performance lay between the durations reported previously for isometric (a few minutes) and whole‐body (half an hour) endurance exercise.

## Abstract 2

Mental fatigue can impair physical performance. We determined the importance of cognitive task type and duration on fatigue and performance. Both memory updating (2‐back) and response inhibition (Stroop) tasks elicited comparable fatigue after 10 min. Subsequent physical performance was impaired after 20–40 min. The fatigue‐performance relationship depended on task duration but not type of executive function.

## 1 INTRODUCTION

Mental fatigue, defined as a psychobiological state that can be caused by engaging in demanding cognitive activity for a prolonged period, is characterized by subjective feelings of tiredness and lack of energy (Marcora et al., 2009). Moreover, it is widely recognized that a state of mental fatigue induced by a cognitive task can impact subsequent endurance exercise performance (for reviews see Brown et al., 2020; Giboin et al., 2019; Van Cutsem et al., 2017). Importantly, meta‐analyses have revealed small‐to‐medium negative effects of performing cognitive tasks on subsequent physical performance (Brown et al., 2020; Giboin et al., 2019). It should be conceded that some studies, albeit a relatively small proportion, have failed to detect effect of mental fatigue on performance. This has occurred for both isometric (Murtagh & Todd, 2004) and whole‐body endurance exercises (Roussey et al., 2018). Additionally, there is evidence that higher levels of motivation can overcome the negative effects of mental fatigue on both cognitive and physical performance (Müller & Apps, 2019).

In a seminal study, Marcora et al. (2009) examined the effects of mental fatigue—induced by a 90‐min AX Continuous Performance Test, which involves multiple executive functions, including attention, error monitoring, memory, and response inhibition—on a subsequent submaximal cycling time to exhaustion test Participants stopped cycling approximately 2‐min earlier when in a state of mental fatigue compared to control, which equated to a 15% reduction in time to exhaustion. This performance deficit was independent of cardiorespiratory activity, with no group differences in exercise‐induced heart rate, stroke volume, cardiac output, mean arterial pressure, oxygen consumption, minute ventilation, and blood lactate. Notably, the mentally fatigued participants started cycling at a higher rating of perceived exertion. Perceived exertion increased at a similar rate during exercise in both groups, and, therefore, the maximal tolerable exertion (and stopping point) was reached sooner by the mentally fatigued participants compared to controls. This finding was replicated by MacMahon et al. (2014) who showed that a 90‐min AX Continuous Performance Test impaired a subsequent 3‐km running time trial. Running times were 2% slower in mentally fatigued participants compared to controls, with no group differences in heart rate, blood lactate, pacing, attention, and perceived exertion.

High‐intensity exercise is unpleasant and exercise‐induced interoceptive signals cue the exerciser to stop and thereby end the discomfort and pain (e.g., Jameson & Ring, 2000). Importantly, the decision to stop exercising is attributable to mental rather than physical factors. With this in mind, researchers (Marcora & Staiano, 2010) have speculated that shorter exercise times following mentally fatiguing cognitive tasks may be due to impaired ability to inhibit responses, such as ignoring signals cuing the exerciser to stop. To evaluate the role of response inhibition processes during a mentally fatiguing cognitive task, Pageaux et al. (2014) examined 5‐km running time trial performance after completing 30‐min incongruent Stroop and congruent Stroop tasks. Both tasks require attention and memory, however, only the incongruent task requires response inhibition (i.e., to stop reading the printed word out loud). Running times were 6% slower following the incongruent Stroop task compared to the congruent Stroop task. This impaired running performance was accompanied by higher mental demand, and concurrent perceived exertion but similar mental fatigue, motivation, pacing, heart rate, and terminal perceived exertion. It was speculated that the elevated perceived exertion was due to increased activity in the anterior cingulate cortex because activity in this brain region has been implicated in the performance of both cognitive and physical tasks (Tanaka et al., 2013), and positively correlated with perceived exertion during exercise tasks (Williamson et al., 2001, 2002).

These data are compatible with the argument that response inhibition during the cognitive task increased perceived exertion and impaired the subsequent bout of exercise. Taken together (MacMahon et al., 2014; Marcora et al., 2009; Pageaux et al., 2014), these findings show that cognitive tasks requiring response inhibition can impair subsequent self‐paced endurance exercise regardless of the state of mental fatigue. Importantly, the evidence indicates that the detrimental effect of mental fatigue on subsequent submaximal whole‐body endurance exercise is more likely to be accounted for by elevated perceived exertion rather than physiological limitations, such as cardiorespiratory and muscle activity (Martin et al., 2018; Pageaux & Lepers, 2016; Van Cutsem et al., 2017) or pacing strategies (Schiphof‐Godart et al., 2018). Accordingly, perception of effort has been deemed the “cardinal exercise stopper” during tasks with fixed demands (Staiano et al., 2018) and the “cardinal exercise regulator” during tasks with variable demands (Graham & Brown, 2021).

Studies indicate that performing a cognitive task impairs subsequent *submaximal* (Bray et al., 2008; Martin et al., 2015) but not *maximal* (Pageaux et al., 2013, 2015; Rozand et al., 2014) muscular contractions. Bray et al. (2008) reported that hold time while performing a 50% maximal voluntary contraction isometric handgrip task was reduced after completing a short (3:40 min) incongruent Stroop task (endurance = 32 s) compared to a congruent Stroop task (endurance = 46 s). The impaired performance was associated with increased forearm electromyographic activity, indicative of a higher neural drive activating muscle motor units to maintain the required force. Similarly, a study examining the dose–response relationship between the duration of an incongruent Stroop task and isometric handgrip exercise at 50% maximum voluntary control found that the cognitive task needed to last at least 4 min to impair subsequent physical task performance (Brown & Bray, 2017).

Moreover, the duration of a response inhibition task was negatively associated with subsequent wall‐sit time; i.e., the longer the cognitive task, the shorter the wall‐sit (Boat et al., 2020). However, a meta‐analysis (Giboin et al., 2019) concluded that prior cognitive task duration was unrelated to subsequent physical task performance. Clearly, evidence is mixed on this dose–response relationship and therefore further investigation is warranted. The evidence reviewed above establishes that a mentally fatiguing cognitive task can impair exercise endurance. Nonetheless, there remain gaps in our understanding of the nature of the relationship between mental fatigue and endurance performance. First, studies have yet to establish the role of cognitive task scheduling on subsequent exercise performance since previous studies have induced mental fatigue using a continuous cognitive task. Second, the importance of response inhibition during the cognitive task has yet to be established. Accordingly, to address these gaps, in the present study we examined the effects of an intermittent series of bouts of cognitive tasks, with and without response inhibition, on mental fatigue and self‐paced rhythmic handgrip exercise.

Our study purposes were threefold. The first study purpose was to assess the effects of a cognitively demanding task (with and without response inhibition) on psychological and physiological indices of mental fatigue as a function of time. The second study purpose was to investigate the effects of cognitive tasks on subsequent performance on a rhythmic handgrip muscular endurance task as a function of time. The third study purpose was to investigate the effects of cognitive tasks on psychological and physiological responses during the endurance task as a function of time. Based on the literature described above, we expected that ratings of mental fatigue would increase and heart rate variability (Ishii et al., 2014; Tanaka et al., 2009) would decrease as a function of time when completing a cognitive task with and without response inhibition. It was expected that a state of mental fatigue, induced by a cognitive task with response inhibition (Stroop) and a cognitive task with memory updating but without response inhibition (N‐back), would similarly impair performance on a subsequent muscular endurance task. However, as no previous research has examined performance on a rhythmic handgrip muscular endurance task, it is difficult to specify the duration of prior cognitive task engagement required to impair performance. Finally, based on previous studies (e.g., MacMahon et al., 2014; Marcora et al., 2009) we expected similar cardio‐respiratory activity as well as increased perceived exertion and muscle activity during exercise.

## 2 METHOD

### 2.1 Participants

Participants were 90 (52 females, 38 males; aged 19.4 ± 1.3 years) undergraduate sport and exercise science students who received course credit for participation. They were asked to abstain from vigorous exercise and alcohol, and to have a regular night's sleep in the 24 h before testing. They were also asked to refrain from eating (1 h) and consuming caffeine (3 h) before testing. The protocol was approved by our Institutional Ethics Committee. Participants provided written informed consent. Power calculations using GPower (Faul et al., 2007) indicated that with a sample size of 90, our study was powered at 80% to detect significant (*p* < .05) between‐within interaction effects (*f* = .124, ${\eta_{p}}^{2}$ = .015) corresponding to a small effect size by analysis of variance (Cohen, 1992).

### 2.2 Design and procedure

The study employed an experimental design with one between‐participant factor (group: Stroop task, 2‐back task, control task) and one within‐participant factor (block: 1, 2, 3, 4). Each block comprised a 10‐min cognitive task followed by a 5‐min physical task. Participants attended one laboratory session. Following an initial briefing, participants were instrumented for physiological measurements. They were asked to sit on a stool throughout and face a computer monitor positioned 1 m away at eye level. After determining the participant's maximal voluntary contraction (MVC) grip force, they were randomly assigned to one of three cognitive task groups. Each group completed four blocks of the 10‐min cognitive task followed by a 5‐min handgrip task. Participants provided ratings before and after each task. They received instruction and completed a 1‐min familiarization of the cognitive task. A £20 retail voucher was offered for the best overall task performance in each group. The experimental protocol is shown in Figure S1 (Supporting Information).

### 2.3 Maximum voluntary contraction

Participants were instructed to squeeze a handgrip as hard as possible for several seconds in order to obtain their MVC (Cooke et al., 2011). They were not aware that their peak force informed the subsequent physical task. A bespoke handgrip dynamometer (Radwin et al., 1991) was held in their dominant hand, placed on their knee, with their arm flexed at approximately 100°. A photograph of the dynamometer is shown in Figure S2 (Supporting Information).

Participants performed a maximal contraction of the handgrip and the peak force was recorded. This was repeated three times, with each contraction separated by a 1‐min rest to allow recovery, with the largest peak force achieved recorded as their maximum voluntary contraction. If the second‐highest peak force was not within 5% of the highest another attempt was required.

### 2.4 Task groups

#### 2.4.1 Control task

Participants watched one of two films about trains (American Orient Express/Venice Simplon Orient Express) from the same documentary series (World Class Trains, Pegasus‐Eagle Rock Entertainment, 2004). Both films are emotionally neutral, elicit stable physiological responses (Silvestrini & Gendolla, 2007), and have been used as control tasks (Marcora et al., 2009; Martin et al., 2015). They watched the first minute as a familiarization task.

#### 2.4.2 2‐back (no response inhibition) cognitive task

The 2‐back task (Braver et al., 1997) activates the anterior cingulate cortex (Tanaka et al., 2014), involves memory updating and attention but does not involve response inhibition (Owen et al., 2005). Participants were shown a continuous series of random consonants: they were required to indicate if the current letter displayed was the same as the one presented two letters earlier. The letters were displayed, once every 2 s for 500 ms, in the center of the monitor. Participants used their non‐dominant hand to press the number 1 key on a keyboard if the current letter displayed was the same as the letter two prior, and the number 2 key if it was different. The task were implemented using E‐Studio (version 2.0.1.97, Psychology Software Tools, Inc., USA). Task performance was assessed by the percentage of correct responses.

#### 2.4.3 Stroop (response inhibition) cognitive task

The incongruent Stroop color word test (Stroop, 1935) activates the anterior cingulate cortex, and involves attention, working memory, and response inhibition (Milham et al., 2003). A series of five color words (red, green, brown, yellow, and blue) were individually displayed in capital letters once every 2 s in the center of the monitor in a different font color to the word meaning. Participants were instructed to verbally name the font color of the word as quickly and accurately as possible. The task was implemented using E‐Studio. If the participant failed to name the correct color of the word whilst it was displayed, stutter, or self‐correct, the response was deemed incorrect. Task performance was assessed by the percentage of correct answers.

### 2.5 Physical task

The physical task (rhythmic handgrip exercise) required participants to hold the handgrip dynamometer in the same position as during the maximum voluntary contraction and to squeeze it with their dominant hand once a second (1 Hz), indicated by an audio metronome, for 5 min. A standardized script was read to participants before the task, at 150 s, and at 270 s, instructing them to *generate as much force as possible in the timeframe for a chance of winning a £20 voucher*. The task time was displayed to participants at 60, 120, 180, 240, and 295 s. Performance was determined by the average peak force as a percentage of MVC per second (force %MVC/s) over the 5‐min task. The force generated per minute as a percentage of total force accumulated over the task was calculated to characterize pacing strategy. A 1‐min familiarization task with visual performance feedback was completed after the maximum voluntary contraction task. Exemplar force trace data alongside the other physiological variables are shown in Figure S3 (Supporting Information).

### 2.6 Physiological measures

All physiological data were acquired via a Power 1401 (Cambridge Electric Design Limited, UK) multi‐channel analog‐to‐digital convertor (16‐bit resolution at a sampling rate of 2.5 kHz) and recorded on a computer running Spike 2 software (version 6.06).

#### 2.6.1 Cardiac responses

Electrocardiographic activity was recorded using silver/silver chloride spot electrodes (Cleartrace, ConMed, USA) attached to the lower left rib, left clavicle, and right clavicle connected to an amplifier (509 cardiac monitor (Morgan, USA). Heart rate and heart rate variability were computed from the R‐R intervals. The root mean square of the successive differences (rMSSD) and the standard deviation (SDNN) of the R‐to‐R wave interval were calculated as time domain surrogates of the high frequency (0.15 to 0.40 Hz) and lower (0.04 to 0.15 Hz) spectral band, respectively. These measures reflect changes in cardiac control via the parasympathetic and combined sympathetic and parasympathetic nervous system, respectively (Cooke et al., 2011).

#### 2.6.2 Muscle activity

The electromyographic activity of the forearm muscles used in gripping, the extensor carpi radialis, and flexor carpi ulnaris, was measured using differential surface electrodes. Following skin preparation (alcohol wipe), they were positioned, alongside a reference electrode, longitudinally on the humorous at approximately 10 cm from the medial epicondyle and 8 cm distal to the lateral epicondyle, respectively (Cooke et al., 2011). Muscle activity was recorded using a Bagnoli‐2 system (Delsys, USA). The signals were rectified, averaged over 30 s, and normalized as a percentage of electromyographic activity at maximum voluntary contraction. Data were lost from seven participants due to noisy recordings during the handgrip task; this is reflected in the reduced degrees of freedom in the reported statistical analyses.

### 2.7 Psychological measures

#### 2.7.1 Fatigue and exertion

The cognitive task was rated immediately following completion for mental exertion and mental fatigue on an 11‐point category ratio (CR‐10) scale. The mental exertion scale was anchored with the extreme descriptors “nothing at all” and “maximal mental exertion”. The mental fatigue scale was anchored with the extreme descriptors “nothing at all” and “totally exhausted”. Participants were reminded that these scales related to mental tiredness and exertion and not physical sensations. Following the tasks, items (exhausted, sleepy, tired, worn‐out) from the fatigue subscale of the profile of mood states were rated on a 5‐point scale, with anchors of 1 “not at all” and 5 “extremely” (Terry et al., 2003). Ratings of perceived exertion were given verbally during the physical tasks at 60, 120, 180, 240, and 295 s on an 11‐point CR‐10 scale (Borg, 1982), anchored with the descriptors “nothing at all” and “maximal”. A task average rating of perceived exertion was calculated from the five ratings. The standard instructions for the scale (Borg, 1982) were read to participants prior to each physical task.

#### 2.7.2 Interest and enjoyment

Task interest and enjoyment were measured using the interest/enjoyment subscale of the intrinsic motivation inventory McAuley et al., 1989). Participants were presented with seven items (e.g., “I enjoyed doing this activity very much”, “I would describe this activity as very interesting”), and responded on a 7‐point scale, with anchors of 1 “not true at all” and 7 “very true.”

### 2.8 Statistical analysis

Statistical analysis was carried out using SPSS 24 software (SPSS: An IBM Company, Chicago, IL, United States). Statistical significance was set at *p* < .05. All data values were expressed as mean ± standard deviation of the mean (*M* ± *SD*) unless otherwise stated. The multivariate solution to ANOVAs has been reported. Partial eta‐squared (${\eta_{p}}^{2}$) was reported as the effect size, with values of 0.02, 0.13, and 0.26 indicating small, medium, and large effects, respectively (Cohen, 1992). Significant ANOVA effects were followed by the least significant difference post hoc tests.

## 3 RESULTS

### 3.1 Cognitive task—Performance

A 2 group (2‐back, Stroop) by 4 block (1, 2, 3, 4) ANOVA on the percentage of correct responses revealed main effects for group, *F*(1, 58) = 62.77, *p* < .001, η^2^ = .52, (*M*_Stroop_ = 98% > *M*_2‐back_ = 88%), and block, *F*(3, 56) = 6.43, *p* = .001, η^2^ = .26, (*M*_1_ = 92% < *M*_2_ = 93% < *M*_3_ = 94% = *M*_4_ = 94%).

### 3.2 Cognitive task—Psychological ratings

**FIGURE 1:**
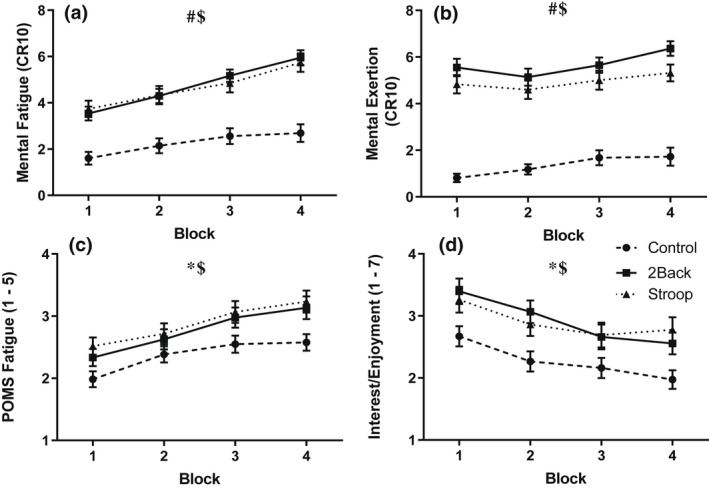
Effects of the cognitive task on mental fatigue (a), mental exertion (b), fatigue (c), and interest/enjoyment (d) as a function of block and group. # (*p* < .001), * (*p* < .05), Significant main effect of group. $ (*p* < .001), Significant main effect of block. Data presented as *M* ± *SEM*.

Ratings of post‐cognitive task mental fatigue, mental exertion, fatigue, and interest/enjoyment are shown in Figure 1. Separate 3 group (control, 2‐back, Stroop) by 4 block (1, 2, 3, 4) ANOVAs revealed group effects for mental fatigue, *F(*2, 87) = 22.31, *p* < .001, η^2^ = .34, mental exertion, *F(*2, 87) = 61.25, *p* < .001, η^2^ = .59, fatigue, *F(*2, 87) = 3.75, *p* = .03, η^2^ = .08, and interest/enjoyment, *F(*2, 87) = 5.14, *p* = .01, η^2^ = .11. Post‐hoc comparisons confirmed that the 2‐back and Stroop groups reported greater fatigue, mental effort, and interest/enjoyment than the control group. The ANOVAs also yielded block effects for mental fatigue, *F*(3, 85) = 29.07, *p* < .001, η^2^ = .51, mental exertion, *F*(3, 85) = 9.60, *p* < .001, η^2^ = .25, fatigue, *F*(3, 85) = 25.48, *p* < .001, η^2^ = .47, and interest/enjoyment, *F*(3, 85) = 16.67, *p* < .001, η^2^ = .37. Ratings of fatigue and effort tended to increase monotonically from the first to the last block of cognitive tasks whilst ratings of and interest/enjoyment tended to increase. Non‐significant group by block interaction effects were noted for mental fatigue, *F*(6, 170) = 22.31, *p* = .08, η^2^ = .06, mental exertion, *F*(6, 170) = 1.80, *p* = .08, η^2^ = .06, fatigue, *F*(6, 170) = 0.93, *p* = .48, η^2^ = .03, and interest/enjoyment, *F*(6, 170) = 1.22, *p* = .30, η^2^ = .41. Finally, it is worth noting that a series of three group ANOVAs confirmed that there were no group differences in pre‐cognitive task (i.e., baseline) mental fatigue, *F(*2, 87) = 1.84, *p* = .16, η^2^ = .04, *M* = 2.10 ± 1.50, and fatigue, *F(*2, 87) = 0.82, *p* = .45, η^2^ = .02, *M* = 1.90 ± 0.80.

### 3.3 Cognitive task—Physiological measures

**FIGURE 2:**
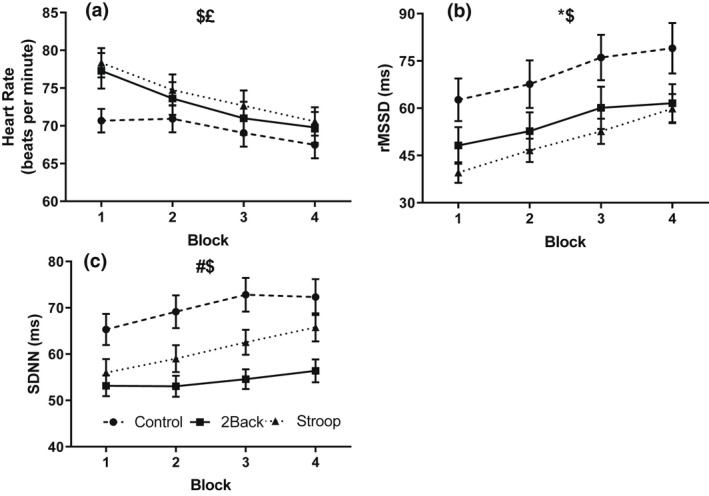
Effects of the cognitive task on heart rate (a), rMSSD (b), and SDNN (c) as a function of block and group. # (*p* < .001), * (*p* < .05), Significant main effect of group. $ (*p* < .001), Significant main effect of block. £ (*p* < .05), Significant group‐by‐block interaction effect. Data presented as *M* ± *SEM*.

Heart rate and heart rate variability (rMSSD, SDNN) during the cognitive tasks (Figure 2) were analyzed with a series of 3 group by 4 block ANOVAs. These analyses yielded group effects for rMSSD, *F(*2, 87) = 3.90, *p* = .02, η^2^ = .08, and SDNN, *F(*2, 87) = 8.41, *p* < .001, η^2^ = .16. Post‐hoc tests confirmed that the 2‐back and Stroop groups exhibited higher heart rate and lower heart rate variability than the control group. The analyses also produced block effects for heart rate, *F(*3, 85) = 33.20, *p* < .001, η^2^ = .54, rMSSD, *F(*3, 85) = 21.75, *p* < .001, η^2^ = .43, and SDNN, *F(*3, 85) = 9.17, *p* < .001, η^2^ = .25. Post‐hoc tests confirmed heart rate slowed, and heart rate variability rose from block 1 to 4. Group by block interaction effects were non‐significant for heart rate, *F(*6, 170) = 1.89, *p* = .08, η^2^ = .06, rMSSD, *F(*6, 170) = 0.59, *p* = .74, η^2^ = .02, and SDNN, *F(*6, 170) = 1.10, *p* = .37, η^2^ = .04.

### 3.4 Physical task—Performance

**FIGURE 3:**
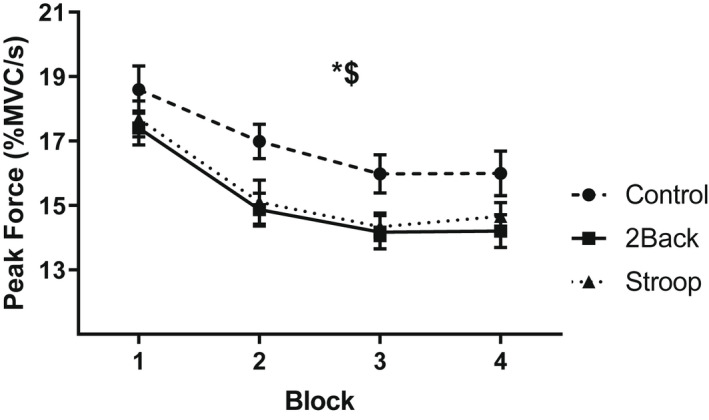
Force production during the handgrip task as a function of block and group. * (*p* < .05) Significant main effect of group. $ (*p* < .001) Significant main effect of block. Data presented as *M* ± *SEM*.

A three group ANOVA confirmed that the maximum grip strength (i.e., MVC) did not differ among the groups, *F(*2, 87) = 0.78, *p* = .46, η^2^ = .02; *M* = 395.82 ± 99.79 N. Physical task performance (Figure 3) was analyzed using a 3 group by 4 block ANOVA on the force produced during rhythmic handgrip exercise, expressed as the average percentage of a participant's MVC per second. This revealed main effects for group, *F(*2, 87) = 3.26, *p* = .04, η^2^ = .07, and block, *F(*3, 85) = 41.97, *p* < .001, η^2^ = .59. Post‐hoc analysis confirmed that the control group (*M* = 16.9, *SD* = 2.7 MVC/s) produced more force than the 2‐back (*M* = 15.2, *SD* = 2.7 MVC/s) and Stroop (*M* = 15.5, *SD* = 2.7 MVC/s) groups. The block by group interaction effect for force production was non‐significant, *F(*6, 170) = 1.22, *p* = .30, η^2^ = .04.

Pacing strategy was analyzed using a 3 group by 4 block by 5 time ANOVA on the percentage of total force produced per minute. This yielded a main effect for time, *F(*4, 84) = 62.92, *p* < .001, η^2^ = .75, and a block‐by‐time interaction effect, *F(*12, 76) = 3.92, *p* < .001, η^2^ = .38; force production declined as the blocks progressed. Importantly, there were no differences in pacing strategy among the groups.

### 3.5 Physical task—Psychological ratings

**FIGURE 4:**
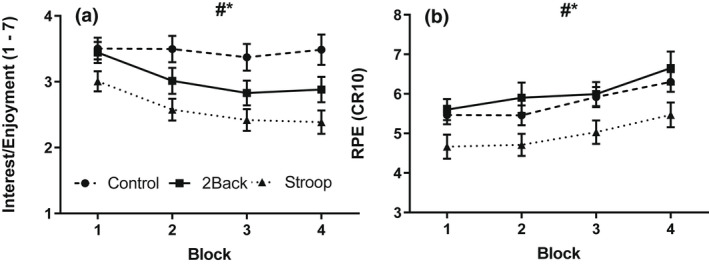
Ratings of interest/enjoyment (a) and perceived exertion (b) during the handgrip task as a function of block and group. # (*p* < .05) Significant main effect of block. * (*p* < .05) Significant main effect of group. Data presented as *M* ± *SEM*.

The interest/enjoyment and perceived exertion ratings for the physical tasks (Figure 4) were analyzed with 3 group by 4 block ANOVAs. Main effects for group, *F(*2, 87) = 6.83, *p* = .002, η^2^ = .14, and block, *F(*3, 85) = 8.90, *p* < .001, η^2^ = .24, were noted for interest/enjoyment, along with a non‐significant interaction effect, *F*(6, 170) = 1.45, *p* = .20, η^2^ = .05. Post hoc tests revealed that the task was less interesting and enjoyable for the Stroop group than the control group. Main effects for group, *F(*2, 87) = 3.54, *p* = .03, η^2^ = .08, and block, *F(*3, 85) = 27.74, *p* < .001, η^2^ = .50, were found for perceived exertion; the Stroop group experienced lower exertion than the control group. Finally, interest and enjoyment decreased whereas exertion increased with repeated task performance.

### 3.6 Physical task ‐ Physiological measures

**FIGURE 5:**
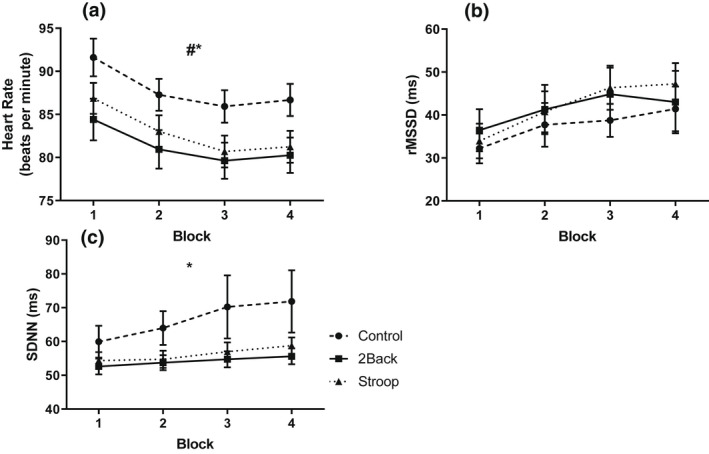
Heart rate (a), rMSSD (b), and SDNN (c) during the handgrip task as a function of block and group. # (*p* < .05) Significant main effect of group. * (*p* < .001) Significant main effect of block. Data presented as *M* ± *SEM*.

**FIGURE 6:**
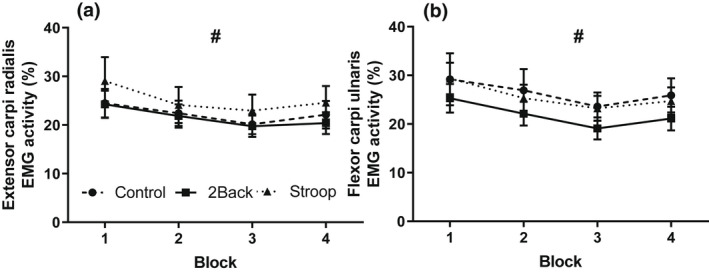
Extensor carpi radialis (a) and flexor carpi ulnaris (b) muscle activity during the handgrip task as a function of block and group. # (*p* < .05) Significant main effect of block. Data presented as *M* ± *SEM*.

Cardiac activity during the physical tasks (Figure 5) was analyzed with a series of 3 group by 4 block ANOVAs. A group main effect was found for heart rate, *F(*2, 87) = 3.15, *p* = .05, η^2^ = .07, which was faster in the control group than the 2‐back group. Block main effects were found for heart rate, *F(*3, 85) = 33.20, *p* < .001, η^2^ = .54, and SDNN, *F(*3, 85) = 13.47, *p* < .001, η^2^ = .32. Heart rate decreased and SDNN increased from block 1 to 3. Muscle activity during the exercise task (Figure 6) was tested using 3 group by 4 block ANOVAs. There were main effects for block for the extensor carpi radialis, *F(*3, 76) = 6.36, *p* < .001, η^2^ = .20, and flexor carpi ulnaris, *F(*2, 78) = 4.58, *p* = .01, η^2^ = .11, forearm muscles which tended to decrease as the blocks progressed. It is worth noting that no group differences were detected in heart rate variability and muscle activity. Finally, no group by block interactions were found.

## 4 DISCUSSION

This is the first study, to our knowledge, to investigate the effects of cognitive task duration and type on subsequent physical muscular endurance. Our study purposes were threefold. First, we assessed the effects of a cognitive task, with and without response inhibition, on indices of mental fatigue as a function of time. Second, we investigated the effects of cognitive task type and duration on subsequent endurance handgrip task. Third, we examined the effects of the cognitive tasks on psychophysiological measures during the endurance handgrip task.

### 4.1 Mental fatigue

The first study purpose was to assess the effects of a cognitively demanding task, with (Stroop) and without (2‐back) response inhibition, on indices of mental fatigue as a function of time. In support of our hypothesis, participants reported greater mental fatigue, general fatigue, and mental exertion following both cognitive tasks relative to watching the documentary film (control). Ratings of mental fatigue increased threefold after completing both 40‐min intermittent cognitive tasks. The magnitude of these increases in fatigue is in line with previous research that assessed mental fatigue following a continuous 45‐min incongruent Stroop task (Smith et al., 2019) and a continuous 120‐min, 2‐back task (Tanaka et al., 2012). These latter studies only assessed fatigue upon task completion and therefore cannot shed light on the evolution of mental fatigue. In contrast, the current study found that mental fatigue increased relative to control after the first task block and continued to increase linearly across the four task blocks, indicating that 10 min of cognitive tasks involving attention and working memory is sufficient to induce a state of mental fatigue. That a similar effect was elicited by both the 2‐back and Stroop tasks indicates that response inhibition during the cognitive task is not necessary to induce mental fatigue. This argument is supported by previous findings demonstrating similar increases in mental fatigue following a 45‐min psychomotor vigilance task, which does not require response inhibition, and following an incongruent Stroop task and AX Continuous Performance Test, which do require response inhibition (Smith et al., 2019). In sum, response inhibition is likely to be a sufficient rather than necessary condition for fatigue‐induced performance detriments. Preliminary evidence that elite athletes perform better on response inhibition tasks than other athletes (Martin et al., 2016) suggests that superior inhibitory control might help resist the negative effects of mental fatigue and thereby contribute to successful endurance performance.

Cardiac activity was measured during the cognitive tasks to evaluate physiological correlates of mental fatigue. Heart rate was higher and heart rate variability lower during the cognitive tasks relative to control, indicative of more mental effort (Mulder, 1992). There were no differences between the 2‐back and Stroop groups, showing that effortful cognitive tasks that elicit changes in cardiac activity are independent of response inhibition processes. The finding that heart rate decreased, and heart rate variability increased as the blocks progressed for all three groups indicates that the impact of mental effort on cardiac activity waned with time. This observation, which is contrary to expectation, is likely to be due to increased task familiarity with exposure. A similar gradual time‐related increase in heart rate variability has been reported in participants who engaged in mentally fatiguing Sudoku puzzles for 120 mins (Gergelyfi et al., 2015). Decreases in parasympathetic and increases in sympathetic activities have been observed by Tanaka and colleagues following one 30‐min block (Tanaka et al., 2009) and four 30‐min blocks (Tanaka et al., 2012) of a 2‐back task. In the single 30‐min block study, heart rate variability measures were averaged over 5‐min epochs and suggested decreased vagal nerve activity after 10 min, which then plateaued for the remaining 20 mins of the 2‐back test. In the current experiment, it is possible that the short breaks between the cognitive tasks, imposed by the physical task (5 min) and self‐report measures (~1 min), in conjunction with increased task familiarity, contributed to the increased heart rate variability as the blocks progressed. Cognitive performance improved as the blocks progressed suggestive of task familiarity. These learning effects could be due to the intermittent blocks of physical and cognitive tasks as opposed to a continuous cognitive task where performance declines (Marcora et al., 2009). Heart rate variability did not change relative to baseline following 45‐min cognitive tasks (Smith et al., 2019), which could represent a transient nature of mental effort and heart rate variability changes. However, when analyzed over 5‐min blocks within the task, the psychomotor vigilance task (i.e., non‐response inhibition) had a higher level of sympathetic activity relative to the response inhibition cognitive tasks. The authors suggest that this could be due to an increased attentional focus due to the demands of the psychomotor vigilance task relative to the Stroop and AX‐CPT response inhibition tasks. The short breaks between presented stimuli in these tasks permit lapses in attentional focus (Ackerman, 2011) and reduced cardiac reactivity. The discrepancy in heart rate variability during the Stroop task between their study and ours could be due to differences in the task. In our study, participants verbally stated the color of the word font rather than indicate the correct answer with a press of a button selected from two possible answers. Verbally stating the word could be more representative of response inhibition as a more natural reaction to a written word is to read and state it rather than press a button. Additionally, in our study all trials were incongruent whereas in their study half of the presented words were incongruent and half were congruent. In sum, there is a relationship between decreased heart rate variability, suggestive of increased mental effort, for cognitive tasks independent of response inhibition, which reduces as a function of time. In relation to our first study purpose, we have shown that engagement in prolonged intermittent cognitive tasks, with and without response inhibition, accentuates the psychological responses and attenuates the physiological responses.

### 4.2 Endurance performance

The second study purpose was to investigate the effects of cognitive task type and duration on subsequent performance on a rhythmic muscular endurance task. As expected, we found that a cognitive task, with or without response inhibition, impaired subsequent muscular endurance performance. This decline in performance was not due to changes in pacing strategy, which was the same for all groups. This finding is consistent with experiments on whole‐body endurance exercise (MacMahon et al., 2014; Pageaux et al., 2014). It is noteworthy that handgrip performance did not deteriorate further over time in the cognitive task groups relative to the control group. This finding shows that mental fatigue impairs subsequent physical performance, but with no further impairments despite increasing mental fatigue. This is the first study to demonstrate a minimum threshold of engagement, of at least 10 min, on a mentally demanding cognitive task to impair performance on a subsequent rhythmic muscular endurance task. In comparison, a previous study reported that a 10‐min Stroop task did not impair subsequent shuttle run performance (Schücker & MacMahon, 2016). The shortest cognitive task duration to impair whole‐body endurance exercise is 30 min (Pageaux et al., 2014), whereas only a few minutes (~4 min) of a cognitive task can impair submaximal isometric exercise (Bray et al., 2008; Brown & Bray, 2017). In relation to our second study purpose, we found that the cognitive task needs to last at least 10 min to impair performance on subsequent rhythmic handgrip exercise. This minimum task duration falls between those reported in the literature for isometric (a few minutes) and whole‐body (half an hour) endurance exercise.

### 4.3 Psychophysiology of endurance

The third study purpose was to investigate the effects of cognitive task type and duration on psychological and physiological measures during the endurance task. Compared to the control group, the cognitive task groups experienced less interest and enjoyment during the physical tasks and more interest and enjoyment during the cognitive tasks. These group differences in interest and enjoyment, an indirect measure of intrinsic motivation (Intrinsic Motivation Inventory, 1994), suggest that the impaired exercise performance of the 2‐back and Stroop groups may have been due to a decline in the desire to perform to the best of their ability. The performance‐related monetary reward we offered, should have increased extrinsic motivation, and could have decreased intrinsic motivation (Deci, 1972), however, this was offered to all participants, and, therefore, it cannot explain the group differences in interest and enjoyment. Participants were instructed to generate as much force as possible during the physical task, which would require maximal effort. The Stroop group reported lower perceived exertion ratings relative to the other two groups, which could be reflective of the lower physical performance relative to the control group and contrasts with our hypothesis and findings from previous experiments where perceived exertion is elevated following response inhibition tasks in time trial performance tasks (MacMahon et al., 2014; Pageaux et al., 2014). Also, contrary to expectation, we observed that heart rate and muscle activity declined over blocks in all groups in proportion to the lower force production. The control group's heart rate was higher during the physical tasks, which reflects their higher force production. The heart rate variability measures were suggestive of reduced mental effort as the blocks progressed. We did not observe elevated muscle activity following cognitive task engagement. This finding is contrary to some previous research with isometric handgrip (Bray et al., 2008) and whole‐body cycling exercise (Pageaux et al., 2015), and could be due to either the intermittent nature of the rhythmic handgrip task and/or the duration of the cognitive tasks.

### 4.4 Study limitations

This study has provided novel evidence concerning the time course of the development of mental fatigue and its effects on muscular endurance performance. The findings should be interpreted in light of a number of possible study limitations. First, the development of mental fatigue, as indexed by psychological and physiological measures, could be attenuated by the intermittent scheduling of the tasks, with a 5‐min physical task after each 10‐min cognitive task. Second, the physical performance deterioration in the 2‐back and Stroop groups could be due to reduced extrinsic motivation as this construct was not measured. However, this is unlikely as monetary reward was offered to the participants to ensure that they were motivated for the tasks. Thirdly, additional physiological measures of effort could have been taken (i.e., pupillometry and electrography) alongside the heart rate variability measures. Finally, the study examined the effects of a cognitive task on rhythmic handgrip exercise performance. Care must be taken when generalizing from a muscular endurance task to whole‐body endurance and isometric muscular endurance tasks.

## 5 CONCLUSION

Our study confirmed that performing a cognitive task for 10 min induces a state of mental fatigue and demonstrated that performing the task for 20 mins or longer will create sufficient mental fatigue to impair subsequent exercise performance. Ours is the first study to confirm this effect in a rhythmic handgrip endurance task. Importantly, we showed that the deleterious effects of a prior cognitive task on endurance were the same for 2‐back and Stroop tasks, indicating that it is not necessary for the cognitive task to involve response inhibition in order to impair endurance exercise performance.

## AUTHOR CONTRIBUTIONS

**Neil Dallaway:** Conceptualization; data curation; formal analysis; investigation; methodology; project administration; software; visualization; writing – original draft; writing – review and editing. **Samuel JE Lucas:** Conceptualization; methodology; project administration; supervision; writing – review and editing. **Christopher Ring:** Conceptualization; data curation; formal analysis; investigation; methodology; project administration; supervision; writing – review and editing.

## Supporting information


**FIGURE S1** Experimental protocol. Arrows indicate timing of ratings questionnaires
**FIGURE S2** Handgrip dynamometer
**FIGURE S3** Sample physiological data from the 5‐min physical task
