# Supplementary material for: Cognitive tasks elicit mental fatigue and impair subsequent physical task endurance: Effects of task duration and type
Source: Psychophysiology. 2022 Jun 21;59(12):e14126. doi: 10.1111/psyp.14126 (PMC9786280; doi:10.1111/psyp.14126)

**SUPPLEMENTARY MATERIALS**

**FIGURE S1** Experimental protocol. Arrows indicate timing of ratings questionnaires.


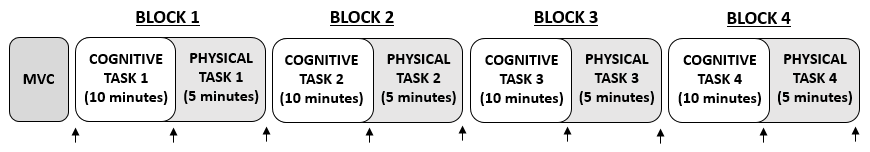


**FIGURE S2** Handgrip dynamometer.


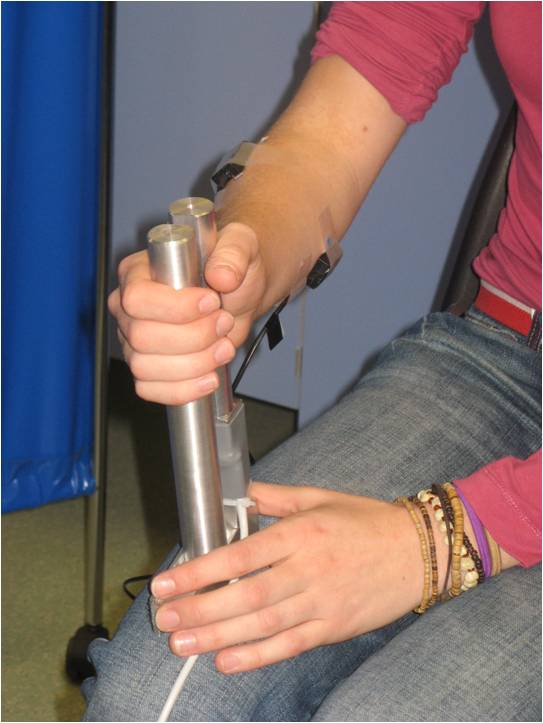


**FIGURE S3** Sample physiological data from the 5-min physical task


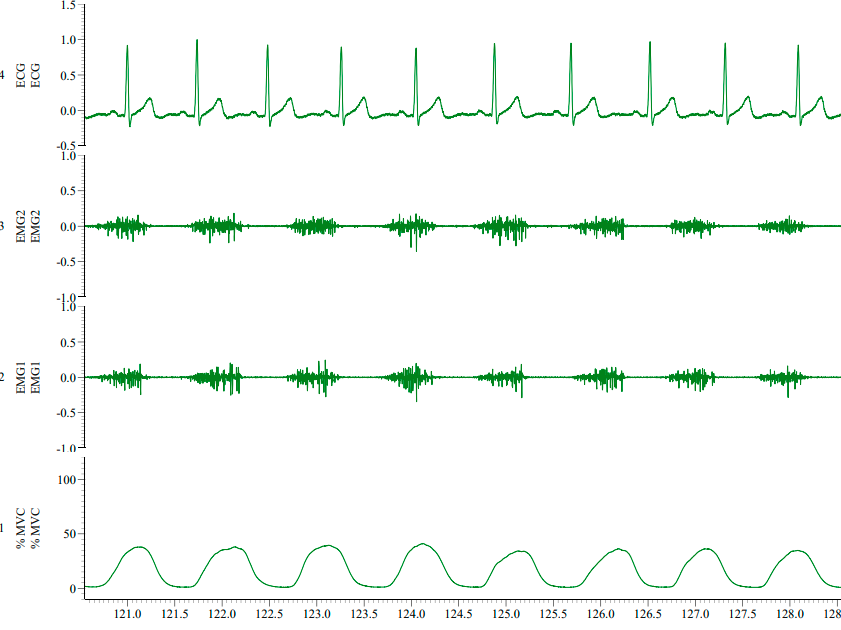

Supplement: Supplementary file 1 — FIGURE S1 Experimental protocol. Arrows indicate timing of ratings questionnaires FIGURE S2 Handgrip dynamometer FIGURE S3 Sample physiological data from the 5‐min physical task [file PSYP-59-e14126-s001.docx]
